# 3D printed teeth with adhesive bridge preparation guide

**DOI:** 10.1038/s41598-024-73433-5

**Published:** 2024-09-24

**Authors:** Michael del Hougne, Greta Behr, Marc Schmitter, Christian Höhne

**Affiliations:** Department of Prosthodontics, University of Würzburg, Pleicherwall 2, 97070 Würzburg, Germany

**Keywords:** Dental education, 3D printing, Clinical practice, Printed tooth, Adhesive bridge preparation, Additive manufacturing, Dental education, Prosthetic dentistry

## Abstract

In this study a 3D printed tooth with adhesive bridge preparation guide was designed and tested for feasibility and evaluated by students. The tooth, printed by a stereolithographic printer, consisted of two differently colored layers with an integrated adhesive bridge preparation. This showed the extent and thickness of the preparation. 42 dental students in the fourth year of their studies were trained in a voluntary course. The printed teeth were evaluated with a questionnaire using German school grades from 1 (best) to 6 (worst). The production of the printed teeth for the adhesive bridge preparation was feasible and inexpensive. Overall, the students rated the teeth as good (Ø1.9 ± 0.2) in the questionnaire and evaluated the teaching method positively in the free text questions. This method supported the students to visualize the target preparation and develop a self-assessment through the ability to control their work directly on their own. The feasibility of this teaching concept was confirmed. It is suitable for teaching of new preparations forms such as adhesive bridges. The color-coded integrated preparation in the printed teeth and the printed tooth model enabled the students to learn the preparation of an adhesive bridge independently.

## Introduction

This manuscript explores the application of 3D-printing in developing practice models specifically for adhesive bridge preparations. Practical courses are an important part of students’ education. Learning new types of preparations is challenging, especially preparations with high technical requirements. The shape of these differs from conventional full coverage crown preparations. An example is the preparation of an adhesive bridge, as they require special preparation techniques to prevent debonding and are considered a minimally invasive treatment approach^1–3^. Different preparation forms are trained at university mainly with standard model teeth from KaVo (KaVo, Biberach an der Riß, Germany) or Frasaco (Frasaco, Tettnang, Germany). There has been a growing demand for more realistic and innovative practice models. 3D printing, which has been used more frequently in the last years, has also found application in the production of practice models in dentistry. 3D-printing represents an additive manufacturing method, accompanied by low production costs and the ability to produce complex forms. Marty et al. designed exercise models for more realistic teaching in pediatric dentistry^4^. The use of 3D printing in dental education has been supported by students and offers teachers more variability. Kröger et al., for example, developed 3D-printed simulation models, which were created from real patient situations^5^. These models were positively evaluated by the students and were a good training for clinical courses. Furthermore, a 3D-printed modular training model, and studies with preclinical students for caries excavation, pulp capping and core build-ups, dentin post preparations and veneer and crown preparations have been reported^6–10^, underlining the feasibility of 3D-printing in dental education.

The aim of this study was to teach students a preparation of an adhesive bridge with printable teeth. Therefore, printed teeth with integrated adhesive bridge preparation were produced. These were tested in a voluntary course with fourth year dental students at university. The acceptance and success of this method was evaluated by the students utilizing a questionnaire.

The hypothesis of this study was, that the printable teeth were an advantage for the students learning, compared to standard model teeth. This method could also be transferred to different preparation forms and represent a cost-effective method.

## Materials and methods

The study and the experimental protocol were approved by the Institutional Review Board of the University of Würzburg (20181116 01) and received an exemption. The usage of irreversibly anonymized data from the questionnaire in the Department of Prosthodontics of the University of Würzburg was granted. All students that participated in the voluntary course were thoroughly informed and signed an informed consent. All methods were performed in accordance with the named guidelines, regulations and Declaration of Helsinki.

### Design of the printed tooth and tooth model

**Fig. 1:**
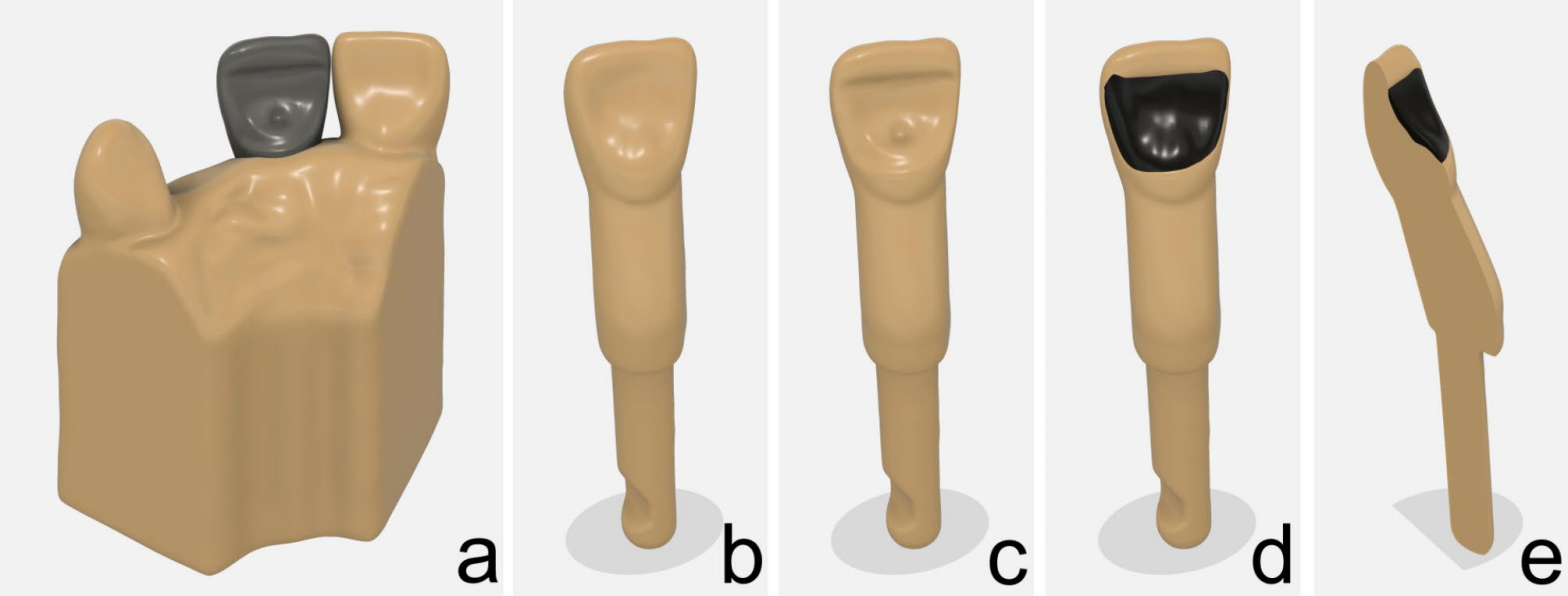
(**a**) The tooth model including the grey tooth with the target adhesive bridge preparation for visualization and control. (**b**) The tooth which corresponds to standard model teeth. (**c**) The ideally prepared tooth. (**d**) The practice tooth with the color-coded integrated preparation. (**e**) A cross section of the practice tooth with the integrated preparation. (Source: own figure).

Initially a standard model tooth of a first incisor (KaVo, Biberach an der Riß, Germany) was scanned with an InEos X5 scanner (Dentsply Sirona, Pennsylvania, USA). Thereupon a preparation for a single retained adhesive bridge of zirconia ceramic was performed. The prepared tooth was also scanned (InEos X5 scanner). The preparation was idealized as a teaching model with the CAD program Autodesk Inventor 2020 (Autodesk, San Rafael, USA) (Fig. 1a). The data in the standard tessellation language (STL) of the two scans were then used to calculate the negative of the preparation (Fig. 1c). A cover in a different color of the preparation was designed to be bonded to the printed tooth (Fig. 1d, e). The surface was identical to the unprepared tooth (Fig. 1b). With the preparation of this tooth the student was able to experience the preparation limits and the amount of the substance removal. As an aid and to show the target preparation, a model was printed with an ideally prepared tooth, which also fits into the standard dental study model (Fig. 1a).

### Production of the teeth

The CAD-designed tooth model was imported into PreForm 3.9 (Formlabs Inc., Somerville, Massachusetts, USA) as a Wavefront OBJ-file. This file was then prepared for 3D printing using a Form2 3D printer (Formlabs Inc.). Dental Model photopolymer resin (RS-F2-DMBE-02, Formlabs Inc.) was used for the tooth model, while Black resin (RS-F2-GPBK-04, Formlabs Inc.) was selected for the preparation cover. The ideally prepared tooth was printed in Grey photopolymer resin (RS-F2-GPGR-04, Formlabs Inc.). In 5 h and 42 min 70 teeth were produced at one build-platform of the printer. The printing time of a single tooth was therefore approximately 5 min. Printing one cover took nearly 2 min. The approximate production of the dental model took 22 min and of the ideally prepared tooth 5 min. The production was completed after washing the prints with 95% isopropanol for 20 min in the Form Wash unit (Formlabs Inc.).

### Completion of the teeth after print

After manufacturing both parts with the 3D printer (Formlabs Inc.), the cover was bonded to the printed tooth with black resin (RS-F2-GPBK-04). In order to obtain optimal material properties, the tooth was hardened in the FormCure unit (Formlabs Inc.) for 60 min. The completion of each tooth took approximately one minute. The material for one tooth costs 0.24 $ and the acquisition costs for the printing equipment a total of 4400 $. The production of the tooth model costs 1.03 $. Thus, the production is cost-effective and efficient.

### Training on the tooth model

**Fig. 2:**
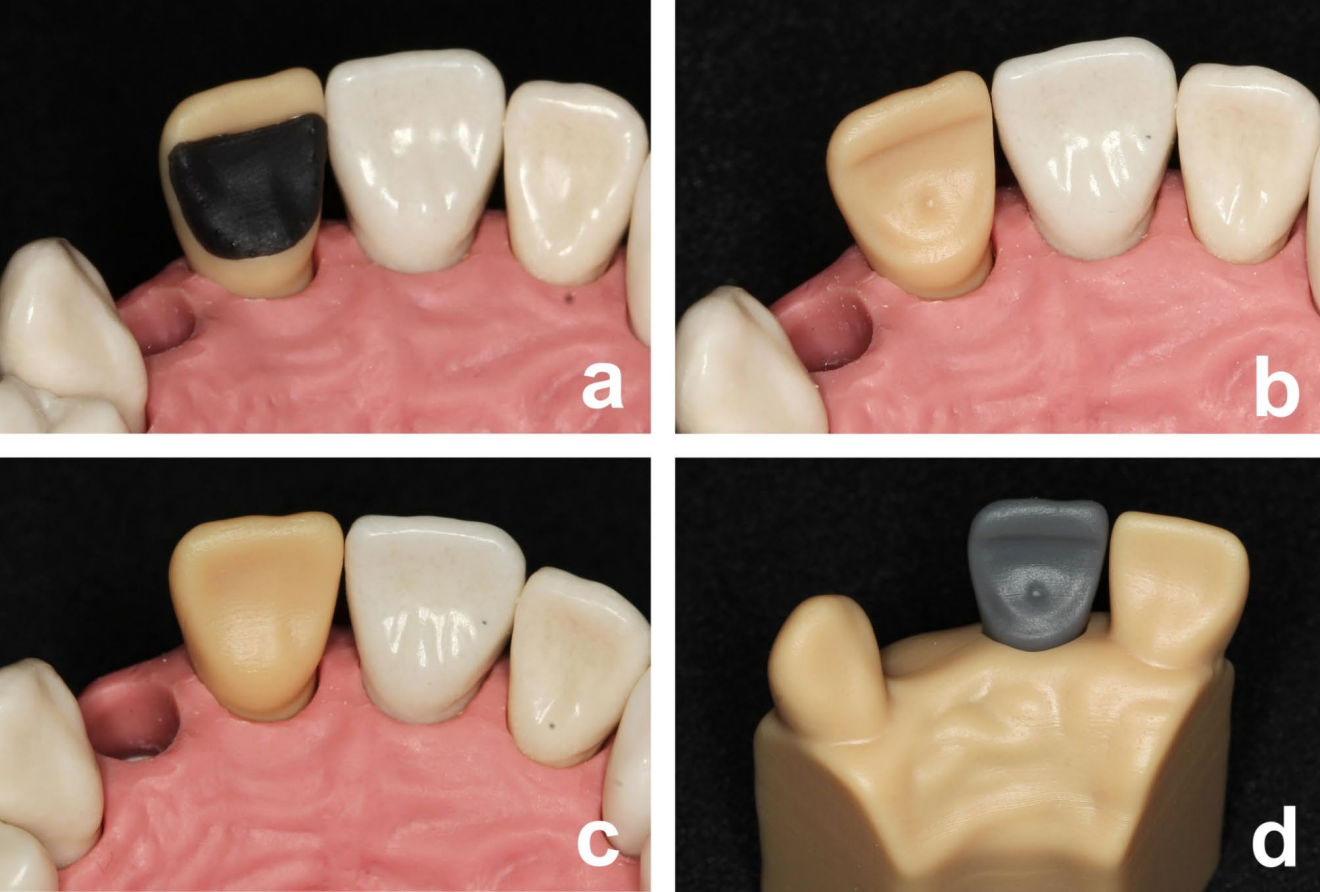
(**a**) The practice tooth mounted into the dental study model. (**b**) The tooth after being ideally prepared. (**c**) The printed standard tooth mounted into the standard dental study model. (**d**) The tooth model including the grey tooth with the target adhesive bridge preparation for visualization and control. (Source: own figure).

In order to test the developed teeth, a voluntary hands-on course was offered for students of the fourth year. This means that the students have not only prepared many model teeth from different manufactures, but also patients’ real teeth. 42 students (23 females and 19 males) from 22 to 50 years participated in the course with 168 teeth. The aim was that the students learn to prepare single retained adhesive bridge of zirconia ceramic with the printed teeth. Already in their preclinical education, students worked with exercise models (Fig. 2a). The target was to stay within the limits of the black resin without eroding or damaging the light brown resin. Figure 2b presents a printed tooth after the preparation and Fig. 2c presents the printed standard tooth mounted into the standard dental study model. The black material can be removed very precisely. Using the Dental model as an illustrative example and to control their success (Fig. 2d) the students removed the black resin of their training tooth completely.

### Questionnaire after practice

To figure out the value of printed teeth for dental education the 42 students participated in a questionnaire (Table 1) after the preparation. The data obtained was irreversibly anonymized. Due to the students’ experience with other model materials and real teeth, they were asked to compare them with the resin of the printed teeth. Using a questionnaire, the students were asked to assess among other things the hardness, the closeness to reality and the practical relevance of the materials with German school grades (1 = Excellent, 2 = Good, 3 = Satisfactory, 4 = Adequate, 5 = Poor, 6 = Unsatisfactory). With the following free text questions, the students were able to express themselves critically about the printed material and its use in teaching courses. EvaSys (Electric Paper Evaluationssysteme GmbH, Lüneburg, Germany) was used to generate the digital evaluation. This type of questionnaire was already tested in a course in endodontic treatment and other printed tooth studies^6–8,10−12^. For this study, the questionnaire was adapted. A single key for the digital questionnaire was given to every student.

**Table 1:** Questionnaire for evaluation of the printed tooth.

1.	Personal Data
1.1 1.2	please enter your gender please enter your age
2.	Comparison of the printed tooth to a standard model tooth
2.1 2.2 2.3	suitable exercise option please enter your age fair examination conditions easy to use
3.	Features of the printed tooth with included preparation
3.1 3.2	the included preparation … … was clearly visible in color … made it easy to get a feeling for the right preparation
4.	Features of the tooth model with the prepared tooth
4.1 4.2 4.3	The color contrast between model and prepared tooth facilitated the recognition of the preparation. The prepared tooth illustrated visually an ideal preparation. The prepared tooth made it easier to control my preparation.
5.	Assessment of the learning results
5.1 5.2 5.3	The learning results were greatest … … with the standard model teeth. … with the printed tooth with built-in preparation … good prepared for the preparation of an adhesive bridge.
6.	Assessment of the learning process
6.1 6.2 6.3 6.4 6.5	The printed tooth had filled me with enthusiasm to improve my skills in the preparation of teeth. The printed tooth with built-in preparation helped me to improve my preparation. The standard model tooth helped me to improve my preparation. With the printed tooth I improve my preparation faster. For my studies I’m interested in more exercises with printed teeth.
7.	Free text questions
7.1 7.2	What could be improved on the printed teeth? In your opinion, what were the advantages of printed teeth in dental education?

### Statistics

Anonymized data was processed utilizing Microsoft Excel 2020 (Microsoft Corporation, Redmond, USA) and IBM SPSS Statistics (Version 25, IBM, Armonk, USA). The questionnaire’s ordinal scaled data was visualized in a bar-chart. The Kolmogorov-Smirnov Test was applied to assess the normality of the data distribution, given its suitability for small sample sizes. For non-normally distributed data, the Mann-Whitney U Test was used to compare individual subgroups. Cronbach’s alpha was calculated to assess the internal consistency of the questionnaire, ensuring the reliability of the measured constructs. The significance level was set at α = 0.05.

## Results

The internal consistency of the questionnaire was nearly excellent, with a Cronbach’s alpha of 0.86. Age and gender of the participants had no significant influence on the results.

### Questions with school grades

**Fig. 3:**
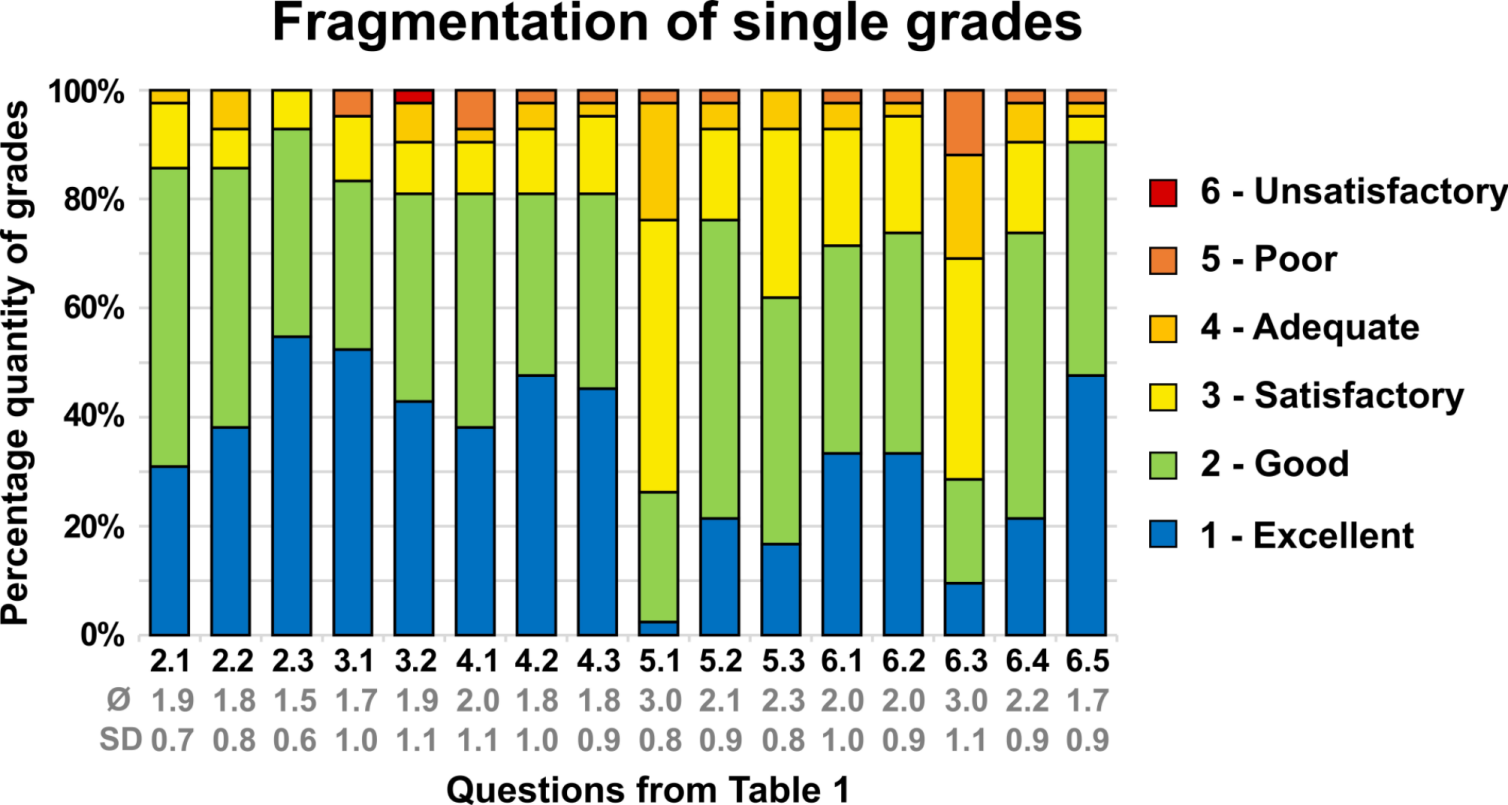
Results of the questionnaire in a stacked barchart (distribution of grades in percentage and mean values quoted below corresponding questions). (Source: own figure).

For the students, the printed teeth were good for practice (Figs. 3–2.1: Ø1.9 ± 0.7) and good for exams (Figs. 3–2.2: Ø1.8 ± 0.8). Students were able to handle them easily (Figs. 3–2.3: Ø1.5 ± 0.6). In the next part of the questionnaire the students gave a general evaluation of the practice teeth. The colored preparation was clearly visible for the students (Figs. 3–3.1: Ø1.7 ± 1.0). The integrated preparation made it easier for the students to get a feeling for the correct preparation (Figs. 3–3.2: Ø1.9 ± 1.1). The evaluation of the tooth model was also positive. The color contrast between the tooth model and the prepared tooth was good (Figs. 3–4.1: Ø2.0 ± 1.1). The model tooth illustrated the target preparation (Figs. 3–4.2: Ø1.8 ± 1.0) and enabled the students to control their own preparation easier (Figs. 3–4.3: Ø1.8 ± 0.9). The students rated their subjective learning success with standard model teeth as satisfactory (Figs. 3–5.1: Ø3.0 ± 0.8), with printed teeth as good (Figs. 3–5.2: Ø2.1 ± 0.9). This finding was significantly different between both groups (*p* = .001). The preparation course with the printed teeth made the students feel well prepared for the preparation of teeth (Figs. 3–5.3: Ø2.3 ± 08). In the last section of the questionnaire the students were asked to evaluate their learning process. The printed teeth aroused enthusiasm in the students to improve their skills in preparing teeth (Figs. 3–6.1: Ø2.0 ± 1.0) and were rated as good (Figs. 3–6.2: Ø2.0 ± 0.9) to improve their preparation skills in contrast to the standard typodont teeth (Figs. 3–6.3: Ø3.0 ± 1.1). This finding was significantly different between both groups (*p* = .001). Also, the preparation was improved faster (Figs. 3–6.4: Ø2.2 ± 0.9) and students wanted more exercises with printed teeth in their studies (Figs. 3–6.5: Ø1.7 ± 0.9).

### Free text questions

In the free text questions, the students made suggestions for improvements and named the advantages of printed teeth from their perspective. Similar answers were combined and counted. As already seen in the evaluation, the students wanted the hardness and color of the practice teeth to resemble the natural tooth structure (hardness: *n* = 14; color: *n* = 7). Some students found the hardness of the resin softer, but still better compared to standard model teeth (*n* = 2). The introduced method for preparing an adhesive bridge was evaluated by the students as a good exercise for learning a new preparation. They said that the necessary layer thickness for the preparation could be better estimated (*n* = 14). Additionally, they would gain a certain independence from the explanations and opinions of the supervisors (*n* = 3). In general, they experienced the use of 3D printing as a possibility to prepare themselves more individually for patients (*n* = 3). Especially the lower costs were a convincing argument for the students to use the printed teeth for further exercises (*n* = 2).

## Discussion

The use of 3D printed teeth for the preparation of adhesive bridges was positively received by students, with an overall rating of 1.9 ± 0.2. This suggests that 3D printing is a promising tool for enhancing dental education.

To produce the printed teeth, the additive manufacturing process of stereolithography was used. While subtractive manufacturing is considered as the gold standard, it is associated with up to 90% waste of materials^13^. With additive manufacturing, components can be manufactured with less material loss. Furthermore, additive manufacturing allows production of objects with more complex forms and with less geometric restrictions^14^. A Form 2 3D printer was used for the printing of the teeth and 3D model. This 3D printer works with a 250-mW laser and a layer thickness of 25–300 μm. In a comparison of 12 3D printers the Form 2 was found to produce reliable and reproducible results with clinically acceptable levels of accuracy^15^. Dental Model photopolymer resin is generally utilized for dental models with high precision and accuracy. Results revealed the desire for improved hardness of the 3D printed teeth. The addition of gypsum powder or Kevlar fibers to resins and alternations in UV curing periods can influence material properties of 3D printed objects^16^. Other printers are available, which print materials of different colors and properties in one step^17^. However, these printers are associated with higher acquisition and material costs and are not available for all academic dental institutions. While 3D printed teeth are accompanied by low running costs, the procedure is time consuming due to several individual production steps.

The positive impact of structured self-assessment on students’ work has been previously confirmed by studies such as those by McDonald and Sharma et al.^18,19^. There are several systems with positive effects on structured self-assessment of preparations, such as CEREC prepCheck (DentsplySirona, York, Pennsylvania, USA) or E4D (Richardson, Texas, USA)^20–23^. Furthermore, virtual reality systems for patient treatment have been developed such as the DentSim Simulator or the IGI (Image Guided Implantology) unit (DenX Ltd, Jerusalem, Israel)^24^. Compared to those systems with high acquisition costs, a 3D printed model is an inexpensive method for learning an ideal target preparation^25^. Printed teeth can be produced for 0.24 $ and the acquisition costs for the printing equipment are a total of 4400 $. A benefit of the 3D printed teeth is an objective (self-)evaluation, by eliminating individual bias of supervisory staff.

As the method presented has only been tested by 42 students at one university, it cannot be generalized for other teaching programs. Small-group hands-on courses are demanding in time and equipment^26^, however, integrating a preparation guide into 3D printed teeth can improve supervision while reducing supervisory staff. Future studies should be conducted to further evaluate the benefits of the objectiveness of this teaching method. While this study utilized self-evaluation by the students, further studies should contextualize these with objective learning outcomes.

Overall, the applied teaching method with 3D printed teeth showed benefits for education.

## Conclusions

The color-coded printed teeth gave the students the possibility to learn the preparation of an adhesive bridge independently and target oriented. The printed teeth gave a good indication of the extend and layer thickness of the preparation and helped the students to develop a feeling for the new preparation. The corresponding tooth model visualized the ideal preparation and enabled the students to compare their own work to the target preparation.

The hypothesis of this study was confirmed. The printed teeth offer an advantage for students to learn new preparation forms, such as an adhesive bridge. The two color-coded integrated preparation and the printed tooth model showed great benefits for dental education.

## Data Availability

All data needed to evaluate the conclusions in the paper are present in the paper.
